# Supplementary material for: Optical spin-state polarization in a binuclear europium complex towards molecule-based coherent light-spin interfaces
Source: Nat Commun. 2021 Apr 12;12:2152. doi: 10.1038/s41467-021-22383-x (PMC8042120; doi:10.1038/s41467-021-22383-x)
Supplement: Supplementary file 1 — Supplementary Information [file 41467_2021_22383_MOESM1_ESM.pdf]

Supplementary Information for

Optical spin-state polarization in a binuclear europium complex towards molecule-based coherent light-spin interfaces

Kuppusamy Senthil Kumar,[1,2]*† Diana Serrano,[3]*† Aline M. Nonat,[4] Benoît Heinrich,[1] Lydia Karmazin,[5] Loïc J. Charbonnière,[4] Philippe Goldner,[3]* and Mario Ruben[2,6,7]*

[1]Institut de Physique et Chimie des Matériaux de Strasbourg (IPCMS), CNRS-Université de Strasbourg, 23, rue du Loess, BP 43, 67034 Strasbourg cedex 2, France.

[2]Institute of Nanotechnology, Karlsruhe Institute of Technology (KIT), Hermann-von-Helmholtz-Platz 1, 76344, Eggenstein-Leopoldshafen, Germany.

[3]Institut de Recherche de Chimie Paris (IRCP), Université PSL, Chimie ParisTech, CNRS, 75005 Paris, France.

[4]Equipe de Synthèse pour l'Analyse, IPHC, UMR 7178, CNRS-Université de Strasbourg, ECPM, 25 rue Becquerel, 67087 Strasbourg Cedex, France.

[5]Service de Radiocristallographie, Fédération de Chimie Le Bel FR2010 CNRS-Université de Strasbourg, 1 rue Blaise Pascal, BP 296/R8, 67008 Strasbourg cedex, France.

[6]Institute for Quantum Materials and Technologies (IQMT), Karlsruhe Institute of Technology (KIT), Hermann-von-Helmholtz-Platz 1, 76344, Eggenstein-Leopoldshafen, Germany.

[7]Université de Strasbourg (Unistra), Institute de Science et d´Ingénierie Supramoléculaire (ISIS), 8, Allée Gaspard Monge, F-67000 Strasbourg, France.

†These two authors have equally contributed.

*e-mail: senthil.kuppusamy2@kit.edu; diana.serrano@chimieparistech.psl.eu; philippe.goldner@chimieparistech.psl.eu; mario.ruben@kit.edu

Contents

1. Supplementary materials 2
2. Supplementary experimental 2
2.1. Sample preparation for spectral hole burning studies 2
2.2. Schematic of experimental setup used for spectral hole burning studies 3
2.3. Preparation of $[Ln_2Cl_6(PicNO)_4(\mu_2\text{-}PicNO)_2]\cdot 2H_2O$ (Ln = Eu ($[Eu_2]$) and Gd ($[Gd_2]$)) complexes 5
2.4. X-ray crystallography 6
3. Supplementary Discussion 7
3.1. X-ray crystal structure analysis of $[Eu_2]$ 7
3.1.1. ORTEP representation and Packing of $[Eu_2]$ in the crystalline state 7

3.1.2 Comparison between the calculated single crystal and powder X-ray diffraction patterns of [Eu_2] 8
3.2. X-ray crystal structure analysis of [Gd_2] 9
3.2.1. ORTEP representation and Packing of [Gd_2] in the crystalline state 10
3.2.2 Comparison between the calculated single crystal and powder X-ray diffraction patterns of [Gd_2] 11
3.3. Supplementary photophysical studies 12
3.3.1. Analysis of photoluminescence characteristics of [Eu_2] 12
3.3.2. Estimation of the triplet energy of 4-picNO from [Gd_2] 15
3.3.3. Analysis of $^7F_0 \rightarrow {}^5D_0$ photoluminescence excitation structure 16
3.3.4. Photostability of [Eu_2] 18
3.3.5. Homogeneous line broadening in [Eu_2] 18
4. Supplementary references 19

1. Supplementary materials

The solvents—ethanol (EtOH; absolute) and ethyl acetate (EtOAc; 99.5%)—used to prepare [$Ln_2Cl_6(PicNO)_4(\mu_2\text{-}PicNO)_2]\cdot 2H_2O$ (Ln = Eu and Gd)—were purchased from VWR chemicals and used as received. Water was distilled from a double distillation setup. $EuCl_3\cdot 6H_2O$ (99.9%; trace metals basis), $GdCl_3\cdot 6H_2O$ (99%; titration), and 4-picoline N-Oxide (98%) were purchased from Sigma-Aldrich and used as received.

2. Supplementary experimental

2.1. Sample preparation for spectral hole burning studies

For LT spectral hole burning measurements, 5 mg of microcrystalline powder sample (Supplementary Figure 1) of [$Eu_2Cl_6(PicNO)_4(\mu_2\text{-}PicNO)_2]\cdot 2H_2O$ ([Eu_2]) was filled into a custom-made sample container (4 mm long x 2 mm wide x 0.5 mm thick) with clear optical openings, as represented in the inset of supplementary Figure 2. This sample container was then introduced into a He bath cryostat. We note that due to the random orientation of the crystallites in the powder sample, no dependency of the molecular complex spectroscopic features (Γ_{inh} and or Γ_h) is expected neither with respect to the laser propagation direction nor polarization.

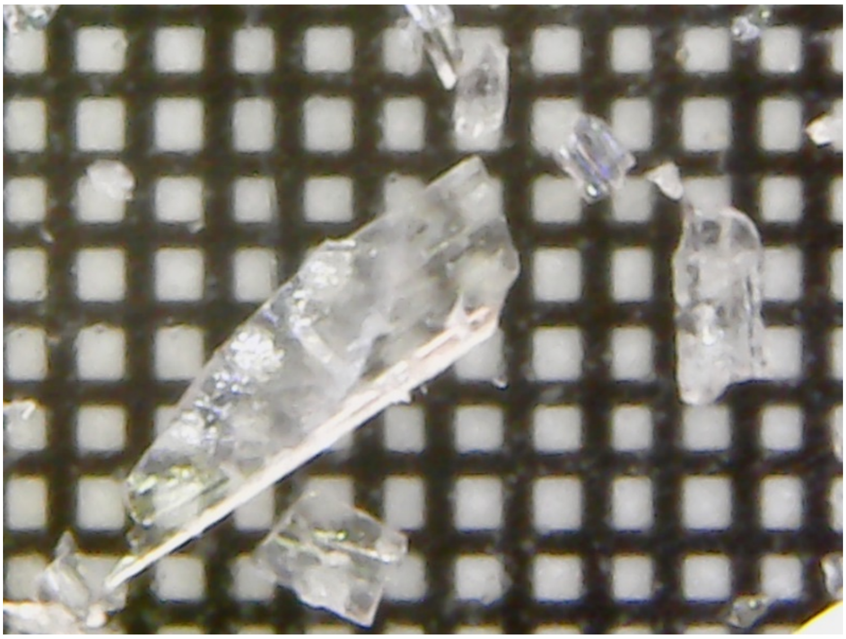

Supplementary Figure 1 | Optical microscopy image of the [Eu_2] microcrystalline powder dispersed over a coverslip. Background grid size = 100 x 100 µm.

2.2. Schematic of experimental setup used for spectral hole burning studies

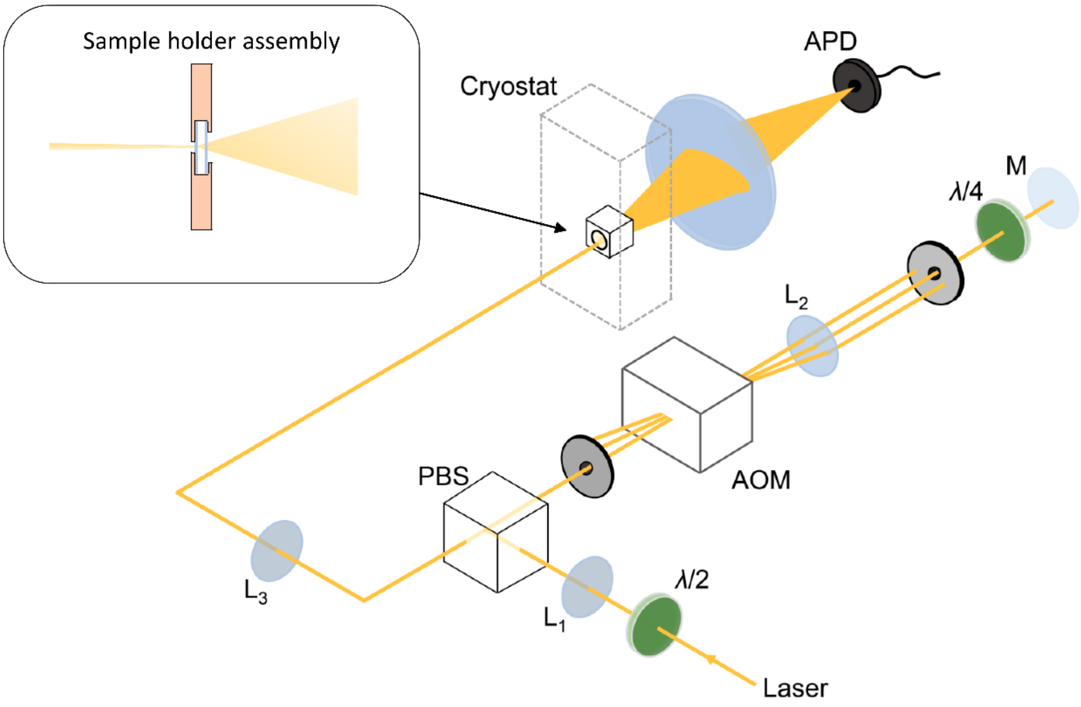

Supplementary Figure 2 | Experimental setup. AOM stands for acousto-optic modulator, PBS for polarizing beam splitter and APD for avalanche photo diode, and M for mirror. L_n (with n=1,2,3) are optical lenses. Unlabeled elements correspond to apertures. Inset: Home-built sample holder assembly. This

figure is partially adapted with permission from *ACS Nano* 2020, 14, 8, 9953–9962. Copyright 2020 American Chemical Society.

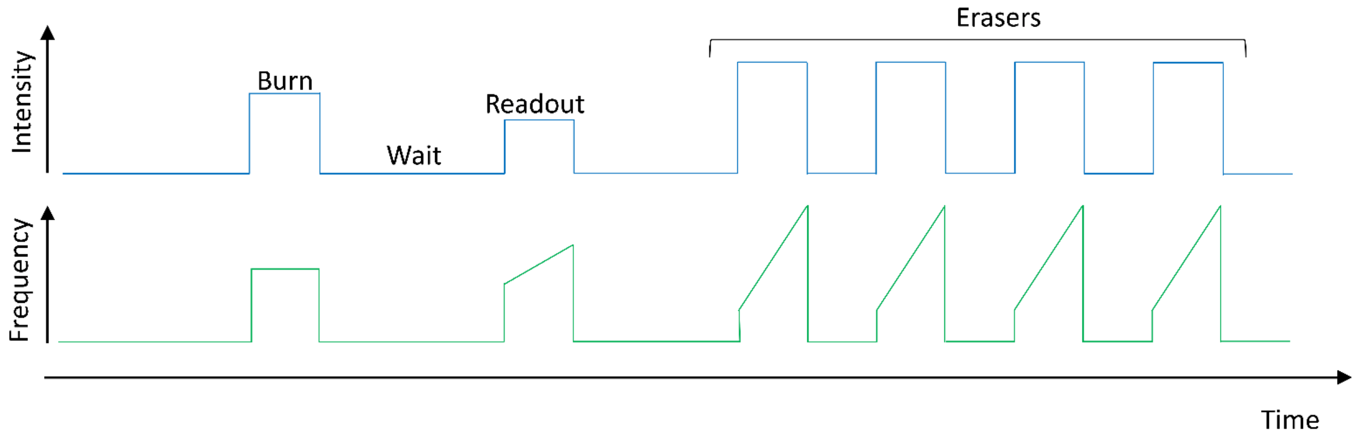

Supplementary Figure 3 | Spectral hole burning pulse sequence. Traces show laser intensity and frequency as a function of time. Burning was maintained for 2 ms and the waiting time before readout was varied from 5 ms to 10 s. The sequence was ended up by a series of hole erasing pulses. See experimental description for more details.

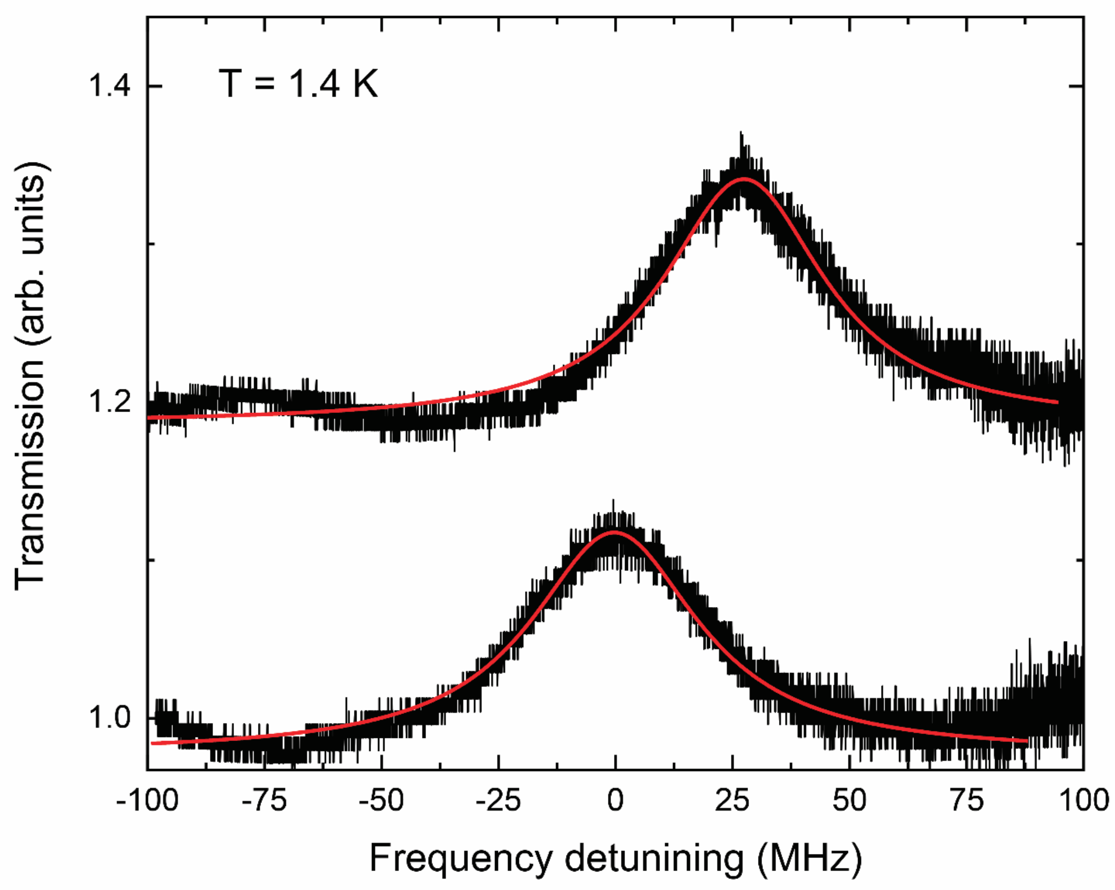

Supplementary Figure 4| Spectral holes burned at two frequencies, separated by 25 MHz, within the optical inhomogeneous transition. As expected, similar hole width is observed independently of the burning frequency. Data are vertically shifted for clarity.

2.3. Preparation of $[Ln_2Cl_6(PicNO)_4(\mu_2\text{-}PicNO)_2]\cdot 2H_2O$ (Ln = Eu ($[Eu_2]$) and Gd ($[Gd_2]$)) complexes

To a solution of 0.35 g (3.2 mmol) of 4-picoline N-Oxide in 12 mL of H_2O, $LnCl_3\cdot 6H_2O$ (Ln = Eu; 0.392 g, 1.07 mmol or Ln = Gd; 0.397 g, 1.07 mmol) was added as a solid (See supplementary Figure 5). The reaction mixture was stirred for 10 min and water was evaporated from the reaction mixture under reduced pressure. The solids were re-dissolved in 15 mL of hot EtOH, filtered, and the filtrate was cooled to RT. About 15-20 mL of EtOAc was carefully added to the filtrate until the formation of a slight precipitate, and the mixture was filtered to obtain a clear solution. The clear solution was left undisturbed for a few days in a closed vial, yielding X-ray quality crystals of $[Ln_2Cl_6(picNO)_4(\mu_2\text{-}picNO)_2]\cdot 2H_2O$ (Ln = Eu or Gd). The crystalline complexes are stable and can be stored and handled at ambient conditions.

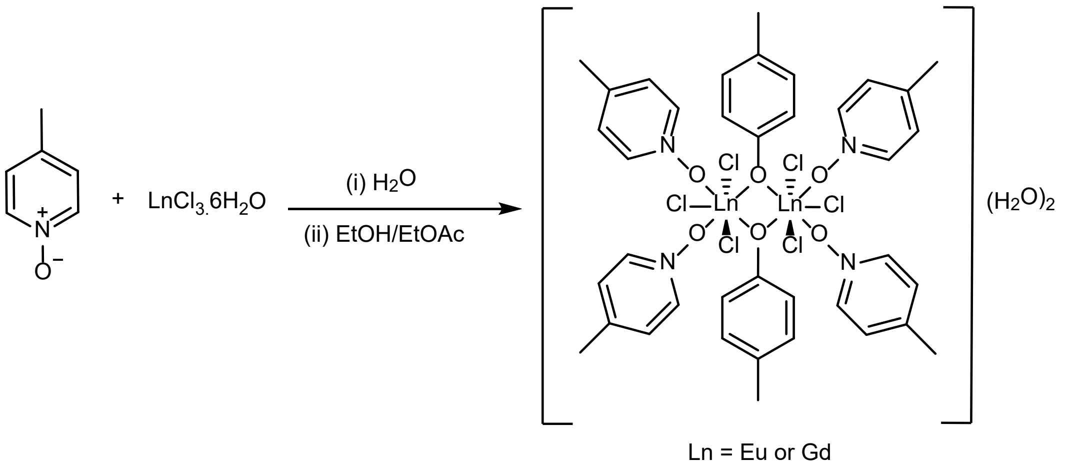

Supplementary Figure 5. Preparation of the molecular complexes $[Eu_2]$ and $[Gd_2]$. Coordination of three anionic Cl^- ligands with each Ln(III) center renders the complex charge neutral. The zwitterionic nature of the 4-picoline N-Oxide ligand is not shown in the complex structure for clarity.

$[Eu_2Cl_6(picNO)_4(\mu_2\text{-}picNO)_2]\cdot 2H_2O$: Elemental analysis (calcd., found for $C_{36}H_{42}Cl_6Eu_2N_6O_6\cdot 2H_2O$): C (35.81, 35.74), H (3.84, 3.84), N (6.96; 6.90). Yield: 0.4 g (61%).

$[Gd_2Cl_6(picNO)_4(\mu_2\text{-}picNO)_2]\cdot 2H_2O$: Elemental analysis (calcd., found for $C_{36}H_{42}Cl_6Gd_2N_6O_6\cdot 2H_2O$): C (35.50, 35.12), H (3.81, 3.79), N (6.90; 6.75). Yield: 0.37 g (57%).

2.4. X-ray crystallography

X-ray diffraction data of [Eu_2] was collected on a Bruker APEX II DUO Kappa-CCD diffractometer equipped with an Oxford Cryosystem liquid N_2 device, using Mo-Kα radiation (λ = 0.71073 Å). The crystal-detector distance was 38 mm. The cell parameters were determined (APEX3 software; M86-EXX229V1 APEX3 User Manual", Bruker AXS Inc., Madison, USA, 2016) from reflections taken from three sets of 12 frames, each at 10 s exposure. The structure was solved using the program SHELXT-2014.[1] The refinement and all further calculations were carried out using SHELXL-2014.[2] Hydrogen atoms were included in calculated positions and treated as riding atoms using SHELXL default parameters. The non-hydrogen atoms were refined anisotropically, using weighted full-matrix least-squares on F^2.

X-ray diffraction data of [Gd_2] complex was collected on a STOE StadiVari 25 diffractometer with a Pilatus300 K detector using GeniX 3D HF micro focus with MoKα radiation (λ = 0.71073 Å). The structure was solved using direct methods and was refined by full-matrix least-squares methods on all F_2 using SHELX-2014 implemented in Olex2. The crystals were mounted on a glass tip using crystallographic oil and placed in a cryostream. Data were collected using φ and ω scans chosen to give a complete asymmetric unit. All non-hydrogen atoms were refined anisotropically. Hydrogen atoms were calculated geometrically riding on their parent atoms.

3. Supplementary Discussion

3.1. X-ray crystal structure analysis of [Eu_2]

3.1.1. ORTEP representation and Packing of [Eu_2] in the crystalline state

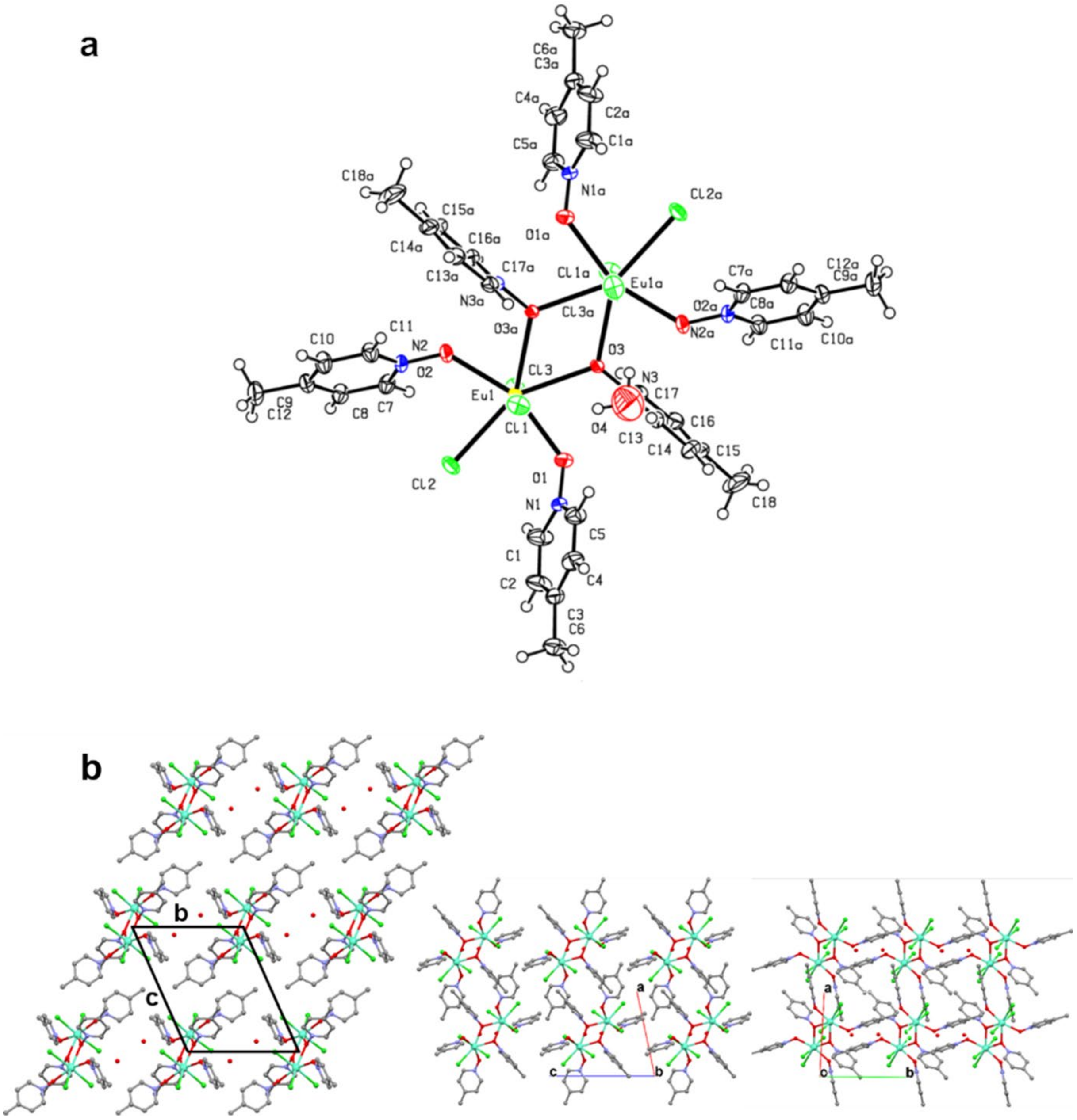

Supplementary Figure 6 | a ORTEP representation of [Eu_2]—The thermal ellipsoids are drawn at a 50% probability level. **b** Arrangement of [Eu_2] in crystalline state at 173 K, as obtained from single crystal XRD (hydrogen atoms not displayed). Left: view of the b×c plane, in which molecules arrange into layers with two co-crystallized water molecules, according to oblique *p*1 lattice (1 molecule per lattice). Successive layers superpose with constant lateral shift leading to single-layer periodicity and triclinic structure with 1 molecule per cell.

Supplementary Table 1. Crystallographic data of [Eu_2]

Formula	$C_{36}H_{42}Cl_6Eu_2N_6O_6{\cdot}2H_2O$	V/Å^3	1181.0(2)
FW/g.mol^{-1}	1207.41	Z	1
T/K	173 K	ρ/mg.m^{-3}	1.698
Crystal System	Triclinic	μ/mm^{-1}	3.022
Space group	P -1	θ min-max/°	1.760 to 30.235
a/Å	9.7938(11)	Reflns collected	54056
b/Å	10.5255(12)	Indep Reflns	6974
c/Å	12.9179(15)	Parameters	265
α/°	66.258(3)	GOF on F^2	1.101
β/°	75.915(4)	R1	0.0436
γ/°	82.000(4)	wR2	0.1292

3.1.2 Comparison between the calculated single crystal and powder X-ray diffraction patterns of [Eu_2]

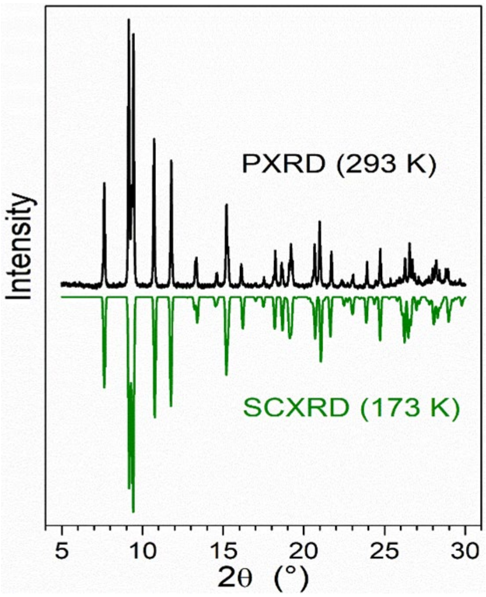

Supplementary Figure 7 | Comparison between the powder X-ray diffraction (PXRD) and the calculated single crystal X-ray diffraction (SCXRD) pattern (inverted trace at bottom) of [Eu_2]. All patterns are composed of same reflections with only tiny changes in peak position and intensity, in relation with tiny variations of lattice parameters and electron densities.

Supplementary Table 2. Structural parameters in single-crystal phase of [Eu_2] at 173 K and bulk crystalline complex at 293 K. Parameters obtained from SCXRD and PXRD data are nearly identical at both temperatures within a volume expansion at the limit of significance (~0.1%) and an unchanged lateral shift between successive layers (~3.19 Å); the tiny $\sqrt{A}/d$ ratio increase (~0.2%) reveals a very slight lateral expansion of layers.

Method	T (K)	Lattice parameters (Å, °), Cell Volume *V* (Å^3) [*Z* = 1 molecule par lattice]<br>b×c sublattice area *A* (Å^2), layer spacing $d = d_{100}$ (Å)
SC-XRD	173	*a* = 9.7938, *b* = 10.5255, *c* = 12.9179, α = 66.258, β = 75.915, γ = 82.000<br>*V* = 1181.02, *A* = 124.46, *d* = 9.4891, $\sqrt{A}/d$ = 1.1757
PXRD	293	*a* = 9.79(32), *b* = 10.52(56), *c* = 12.90(85), α = 66.57, β = 75.65, γ = 82.29<br>*V* = 1181.84, *A* = 124.6(7), *d* = 9.48, $\sqrt{A}/d$ = 1.178

3.2. X-ray crystal structure analysis of [Gd_2]

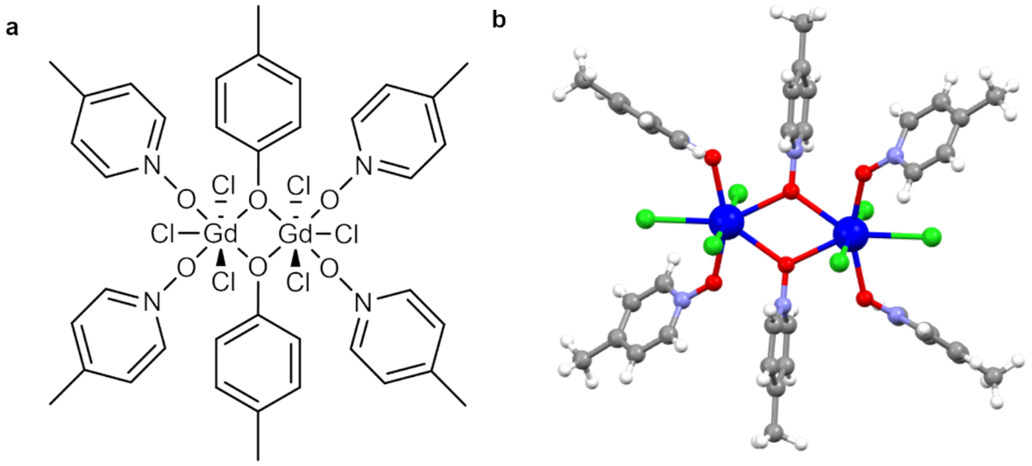

Supplementary Figure 8 | Structure of [Gd_2]. **a** Molecular structure of the complex showing the ligands coordinating with the Gd(III) centers. **b** X-ray crystal structure of the complex. Coordination geometry around each Eu(III) centre of the complex is best described as pentagonal bipyramidal. The co-crystallized water molecules are omitted for clarity. Colour code: H, white; C, grey; N, blue; Cl, green; Gd, blue.

3.2.1. ORTEP representation and Packing of $[Gd_2]$ in the crystalline state

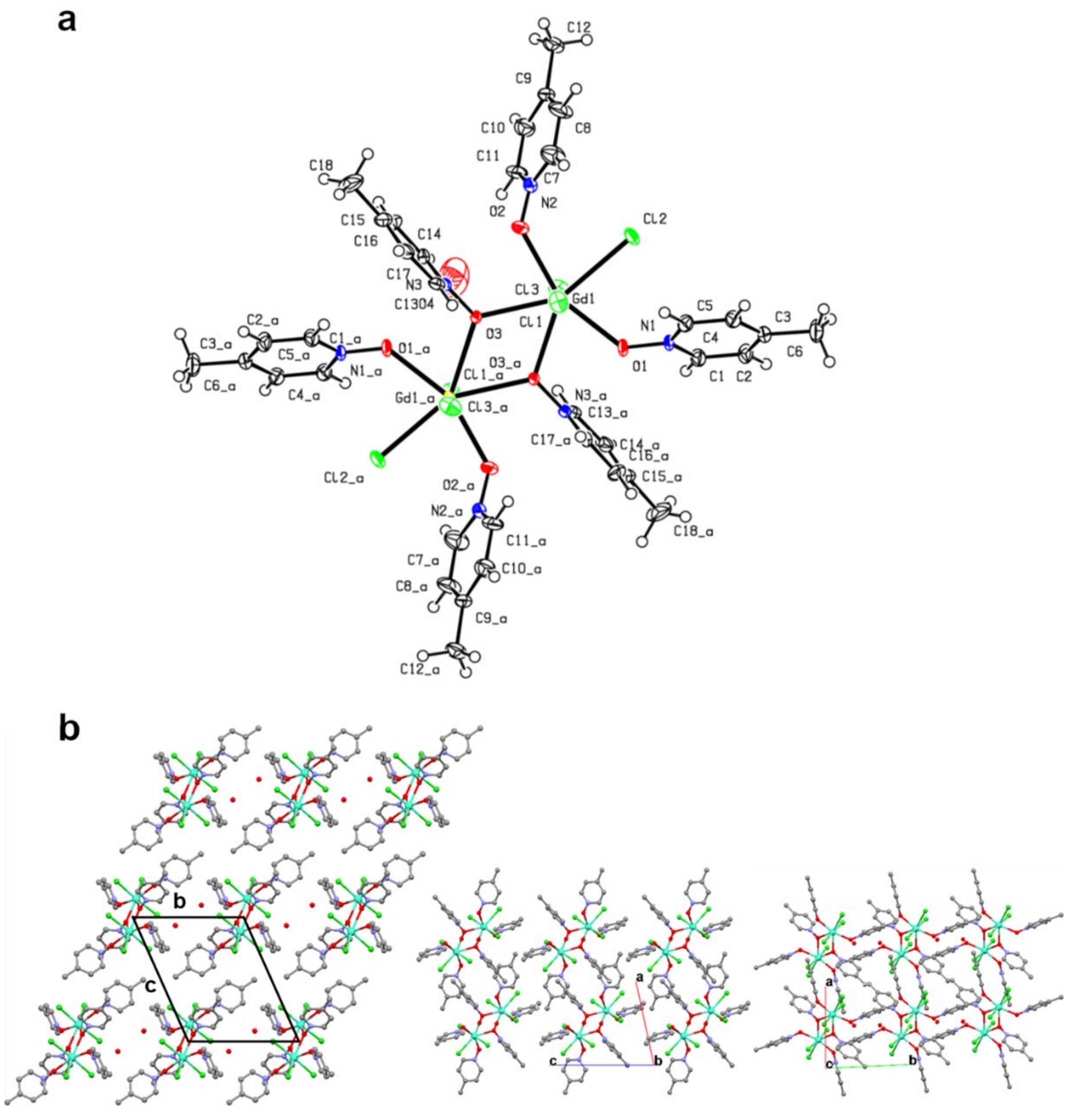

Supplementary Figure 9 | a ORTEP representation of $[Gd_2]$—The thermal ellipsoids are drawn at a 50% probability level. **b** Arrangement of $[Gd_2]$ in crystalline state at 180 K, as obtained from single-crystal XRD (hydrogen atoms not displayed). Left: view of the b×c plane, in which molecules arrange into layers with two co-crystallized water molecules, according to oblique *p*1 lattice (1 molecule per lattice). Successive layers superpose with constant lateral shift leading to single-layer periodicity and triclinic structure with 1 molecule par cell.

Supplementary Table 3. Crystallographic data of [Gd_2]

Formula	$C_{36}H_{42}Cl_6Gd_2N_6O_6 \cdot 2H_2O$	V/Å^3	1166.85
FW/g.mol^{-1}	1213.96	Z	1
T/K	180 K	ρ/mg.m^{-3}	1.728
Crystal System	Triclinic	μ/mm^{-1}	3.213
Space group	P -1	θ min-max/°	2.9370-27.8190
a/Å	9.7768	Reflns collected	13777
b/Å	10.4291	Indep Reflns	5505
c/Å	12.8331	Parameters	265
α/°	66.978(5)	GOF on F^2	1.105
β/°	75.885(5)	R1	0.0330
γ/°	82.310(5)	wR2	0.0880

3.2.2 Comparison between the calculated single crystal and powder X-ray diffraction patterns of [Gd_2]

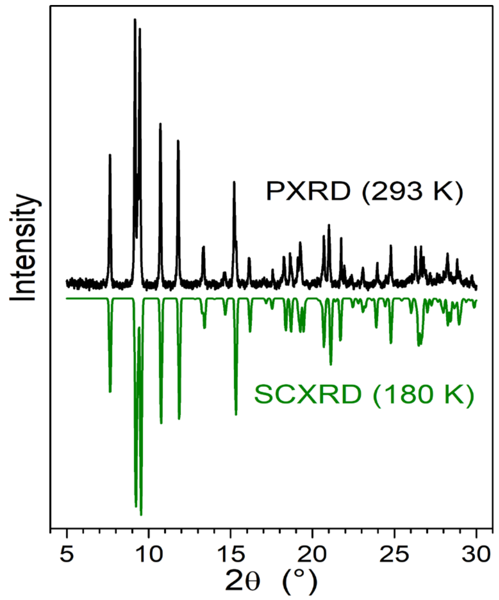

Supplementary Figure 10| Comparison between the powder X-ray diffraction (PXRD) and the calculated single crystal X-ray diffraction (SCXRD) pattern (inverted trace at bottom) of [Gd_2]. All patterns are composed of same reflections with only tiny changes in peak position and intensity, in relation with tiny variations of lattice parameters and electron densities.

Supplementary Table S4. Structural parameters in single-crystal phase of [Gd_2] at 173 K and bulk crystalline complex at 293 K. Parameters obtained from SCXRD and PXRD data are nearly identical at both temperatures within a volume expansion at the limit of significance (~0.1%) and an unchanged lateral shift between successive layers (~3.19 Å); the tiny $\sqrt{A}/d$ ratio increase (~0.2%) reveals a very slight lateral expansion of layers.

Method	T (K)	Lattice parameters (Å, °), Cell Volume V (Å^3) [Z = 1 molecule par lattice]<br>b×c sublattice area A (Å^2), layer spacing $d = d_{100}$ (Å)
SC-XRD	180	a = 9.7768, b = 10.4291, c = 12.8331, α = 66.978, β = 75.885, γ = 82.310<br>V = 1166.22, A = 123.14, d = 9.4712, $\sqrt{A}/d$ = 1.1716
PXRD	293	a = 9.78(63), b = 10.51(23), c = 12.88(72), α = 66.60, β = 75.57, γ = 82.27<br>V = 1177.40, A = 124.3(3), d = 9.47, $\sqrt{A}/d$ = 1.177

3.3. Supplementary photophysical studies

3.3.1. Analysis of photoluminescence characteristics of [Eu_2]

In a continuation of the discussion in the main script, an analysis of the PL characteristics of [Eu_2] is presented in the following sections. The near infra-red (NIR) $^5D_0 \rightarrow {}^7F_{5,6}$ transitions were seen around 745 nm ($^5D_0 \rightarrow {}^7F_5$) and in the 801 nm to 836 nm ($^5D_0 \rightarrow {}^7F_6$) region. The $^5D_0 \rightarrow {}^7F_5$ and $^5D_0 \rightarrow {}^7F_6$ transitions amount to 3.3% and 7.5%, respectively, of the total emission intensity. Remarkably, the $^5D_0 \rightarrow {}^7F_4$ transition centered around 700 nm accounts for 26% of the total emission. Overall, a deep red emission with chromaticity coordinates (x = 0.6615, y = 0.3382) on the commission internationale de l'éclairage (CIE) color space (Supplementary Figure 11a) was observed for the complex. A strictly mono-exponential decay, confirming the presence of a single emitting Eu(III) species, of luminescence was obtained upon monitoring decay at 616 nm (Supplementary Figure 11b).

It is well documented that the $^5D_0 \rightarrow {}^7F_{2,4,6}$ transitions are allowed electric-dipole transitions, which are strongly influenced by the crystal field. The emission spectrum of the [Eu_2] is in agreement with the low coordination symmetry of the complex, as determined from the X-ray crystallography study. First of all, the $^5D_0 \rightarrow {}^7F_0$ transition, which is essential for SHB in the present studies, is unambiguously seen at 580 nm. In addition, the degree of distortion around Eu(III)—as evaluated from the measurement of the intensity ratio $I(^5D_0 \rightarrow {}^7F_2)/I(^5D_0 \rightarrow {}^7F_1)$—was found to be R_{21} = 7.6 and reveals a distorted coordination

environment or low-site symmetry around the Eu(III) centers in the complex. The rationale behind this approach is that the magnetic dipolar $^5D_0 \rightarrow ^7F_1$ transition is not influenced by the crystal field, whereas the electric dipolar $^5D_0 \rightarrow ^7F_2$ transition is strongly influenced (hypersensitive) by the local symmetry of the electric/crystal field around a Eu(III) center. Finally, the intensity ratio $I(^5D_0 \rightarrow ^7F_4)/I(^5D_0 \rightarrow ^7F_2)$ is smaller than one, as expected in a low symmetry environment. A significant contribution of the $^5D_0 \rightarrow ^7F_4$ in [Eu_2] is not unusual for R-PyNO-based Eu(III) complexes, as reported in the literature before.[3] This is due to the high polarizability of R-PyNO ligands, bringing a high contribution of the ligand polarizability-dependent dynamic coupling mechanism (*versus* electric dipole mechanism) according to Judd-Ofelt theory.

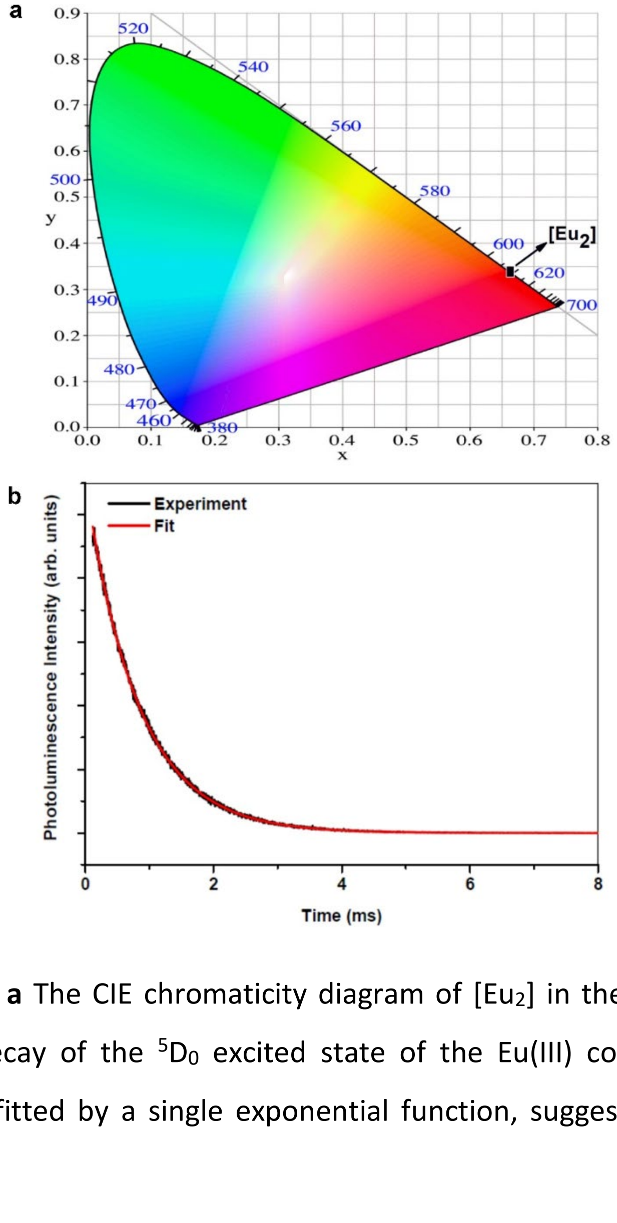

Supplementary Figure 11 | a The CIE chromaticity diagram of [Eu_2] in the solid state: x = 0.6615, y = 0.3382. **b** Luminescence decay of the $^5D_0$ excited state of the Eu(III) complex measured at RT, the fluorescence decay is well fitted by a single exponential function, suggesting a single emitting Eu(III) centre.

A PL quantum yield of 38±6% was experimentally determined for the complex. The total PL quantum yield, Q_{tot}, can be represented as the product of the sensitization efficiency (η_{sens}) of the ligand and the intrinsic quantum yield (Q_{Eu}) of the europium emission from the $^5D_0$ level:

$Q_{tot} = \eta_{sens} \times Q_{Eu}$ (1)

$Q_{Eu} = \tau_{obs}/\tau_{rad}$ (2)

Where τ_{rad} is the radiative lifetime of the $^5D_0$ level. The radiative lifetime is expressed as

$\tau_{rad} = [A_{MD} \times (n)^3 \times I_{tot}/I_{MD}]^{-1}$ (3)

Where A_{MD} is the spontaneous emission probability for the $^5D_0 \rightarrow {}^7F_1$ transition in vacuum (taken here as 14.65 s^{-1}), n is the refractive index (taken here as 1.5) of the solid complex, and I_{tot}/I_{MD} is the ratio of the total integrated emission intensity (for all transitions from the $^5D_0$ state to the ^7F_J manifold (I_{tot})) to that of the magnetic dipole $^5D_0 \rightarrow {}^7F_1$ transition (I_{MD}); I_{tot}/I_{MD} = 14.95 is obtained from the spectrum depicted in the main text, see Figure 2b. The values of τ_{rad} = 1353 µs and η_{sens} = ~62% were calculated from equations 3 and 1, respectively. An intrinsic Eu quantum yield (Q_{Eu}) = ~61% was obtained from equation 2.

The obtained values of τ_{rad} and τ_{obs} are used to estimate the competition between the radiative and nonradiative processes, which in turn determines Q_{Eu}. While τ_{rad} is not influenced by the non-radiative processes, such processes influence τ_{obs}. Thus, the rate constants for radiative (k_r) and non-radiative (k_{nr}) processes can be estimated from equations 4 and 5.

$k_r = 1/\tau_{rad}$ (4)

and

$k_{nr} = [(1/\tau_{obs}) - (k_r)]$ (5)

The obtained k_r = 739 S^{-1} and k_{nr} = 477 S^{-1} indicate the role of competitive non-radiative relaxation process—most probably, C-H vibrations arising from the 4-picNO ligand skeletons—in reducing Q_{Eu}.

Apart from Q_{Eu}, the total sensitization (energy transfer) efficiency (η_{sens}) of ligand also plays a crucial role in determining the overall luminescence quantum yield (Q_{tot}) of Eu(III) luminescence upon excitation of the light absorbing ligand. The sensitization efficiency of a ligand depends on the energy separation (ΔE) between the triplet state (E_T) of the ligand and Eu(III) acceptor state ($^5D_0$). An energy gap of 2500 cm^{-1} < $\Delta E(^3\pi\pi - {}^5D_0)$ < 3500 cm^{-1} is proposed optimal for the luminescence sensitization of Eu(III). A non-optimal ΔE = ~6579 cm^{-1} has been estimated for [Eu_2], which justifies the η_{sens} = ~62.54% calculated for 4-picNO.

Note, E_T = 23809 cm^{-1} has been estimated for 4-picNO (see supplementary section 3.3.2 and supplementary Figure 12) from the phosphorescence emission of [Gd_2] at 77 K.

Despite the presence of non-radiative pathways and non-optimal ΔE, [Eu_2] complex featured ~38±6% total luminescence quantum yield. A sensitization mechanism involving direct funnelling of energy from the triplet level of 4-PicNO to ^5D_J (J = 2, 1, and 0) levels and subsequent radiative relaxation of the $^5D_0$ level to ground-state J multiplets (Fig. 2a) caused the Eu(III) centred emission in [Eu_2].

The optimal energy gap of 2500 cm^{-1} < $\Delta E(^3\pi\pi - ^5D_0)$ < 3500 cm^{-1} required for the efficient sensitization of Eu(III) luminescence could be achieved by reducing $^3\pi\pi$ energy of the 4-picNO ligand by replacing the methyl group with an electron withdrawing group; for example, CCl_3.

3.3.2. Estimation of the triplet energy of 4-picNO from [Gd_2]

To assess the triplet state energy of the 4-picNO ligand involved in the Eu(III) luminescence sensitization process, PL spectra of [Gd_2] was measured upon excitation (λ_{ex}) of 4-picNO at 291 nm. A broad and structured emission band with λ_{max} = 492 nm was observed (Supplementary Figure 12). By considering the lowest energy fine structure located at ~420 nm, a triplet energy *(E_T)* = 23809 cm^{-1} is obtained for 4-picNO, which is in agreement with the 0-phonon component determined from the intersection of the x axis and the tangent line at $I_{max/2}$. The $^3\pi\pi^*$ excited state decayed mono-exponentially with a lifetime of 1.08 ms.

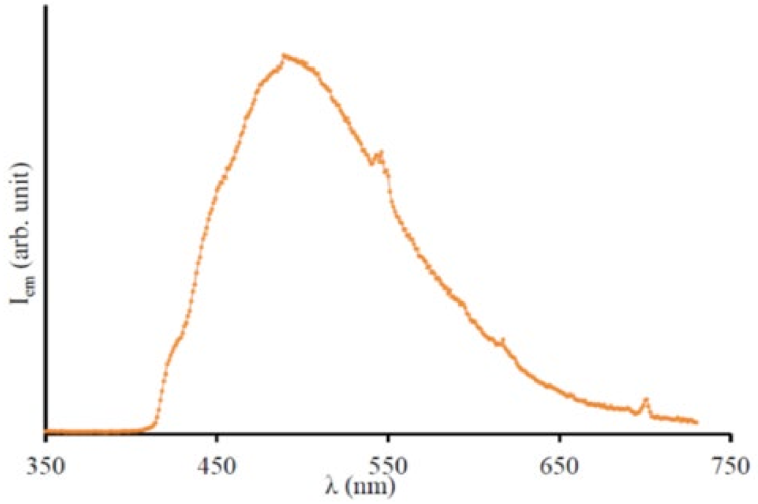

Supplementary Figure 12 | Emission spectra of [Gd_2] in the solid state at 77 K (λ_{ex} = 291 nm). The small intensity bands observed above 475 nm are most probably due to Tb(III) and Eu(III) contamination present in the $GdCl_3{\cdot}6H_2O$ used to prepare the Gd-complex. Efficient sensitization of Eu(III) and Tb(III) luminescence by 4-picNO caused the appearance of Tb(III)- and Eu(III)-based bands, despite their small concentrations (~1%).

3.3.3. Analysis of $^7F_0 \rightarrow {}^5D_0$ photoluminescence excitation structure

As shown and discussed in the main manuscript, the low temperature excitation spectrum of the $^7F_0 \rightarrow {}^5D_0$ Eu(III) transition show a main absorption peak at 580.185 nm (about 50 GHz FWHM) surrounded by several partially resolved side peaks (Supplementary Figure 13a). To shed light on the origin of the side peaks, emission spectra and photoluminescence decay curves were recorded under selective laser excitation of the peaks. Clear differences were observed in emission peaks relative intensities (Supplementary Figure 13b) and fluorescence lifetimes (Supplementary Figure 13c), indicating that Eu(III) ions are found in different crystalline sub-sites. These sub-sites are most likely due to defects and/or minor configurational modifications in the Eu(III) local environment. The single exponential decay curves obtained from all sub-sites indicate no energy transfer or interaction between them.

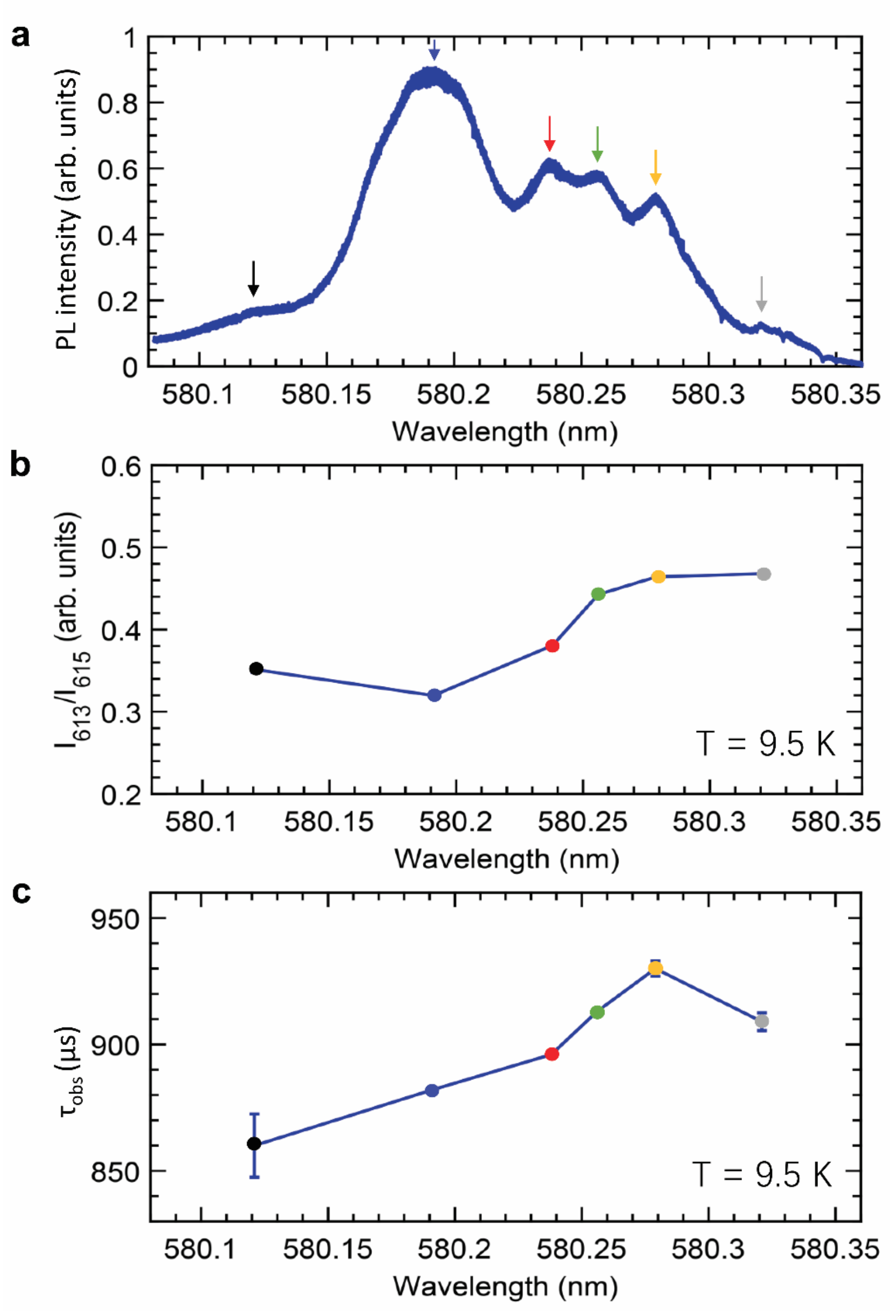

Supplementary Figure 13 | Low-temperature high-resolution PL spectroscopy. **a** Excitation spectrum for the Eu(III): $^7F_0 \rightarrow {}^5D_0$ transition obtained by monitoring the $^5D_0 \rightarrow {}^7F_J$ (J=2-6) Eu(III) emissions (see methods in main manuscript for further details). Colored arrows indicate the spectral positions at which we recorded emission spectra (**b**) and PL decays (**c**). **b** Intensity ratio between the 613 nm and the 615 nm emission peaks (associated to transitions from the $^5D_0$ singlet towards the $^7F_2$ multiplet, see Figure 2b in the main script), as a function of $^7F_0 \rightarrow {}^5D_0$ excitation wavelength. The observed emission branching ratio variation indicates the selective excitation of different Eu(III) sub-sites. **c** Fluorescence lifetime as a function of $^7F_0 \rightarrow {}^5D_0$ excitation wavelength. Lifetime values were obtained by single exponential fit to the $^5D_0 \rightarrow {}^7F_J$ (J=2-6) emissions decay curves (see methods in main manuscript for further details).

3.3.4. Photostability of [Eu_2]

Photostability of a molecular material is an important aspect determining the QIP utility of the material. Absence of photobleaching in the [Eu_2] complex was confirmed by monitoring the Eu(III) photoluminescence intensity under different excitation powers and different temperatures (Supplementary Fig 14). Furthermore, no signs of degradation of the photophysical properties of the complex were observed after repeated experiments on the same sample. This reflects the very stable charge state of Eu(III).[4]

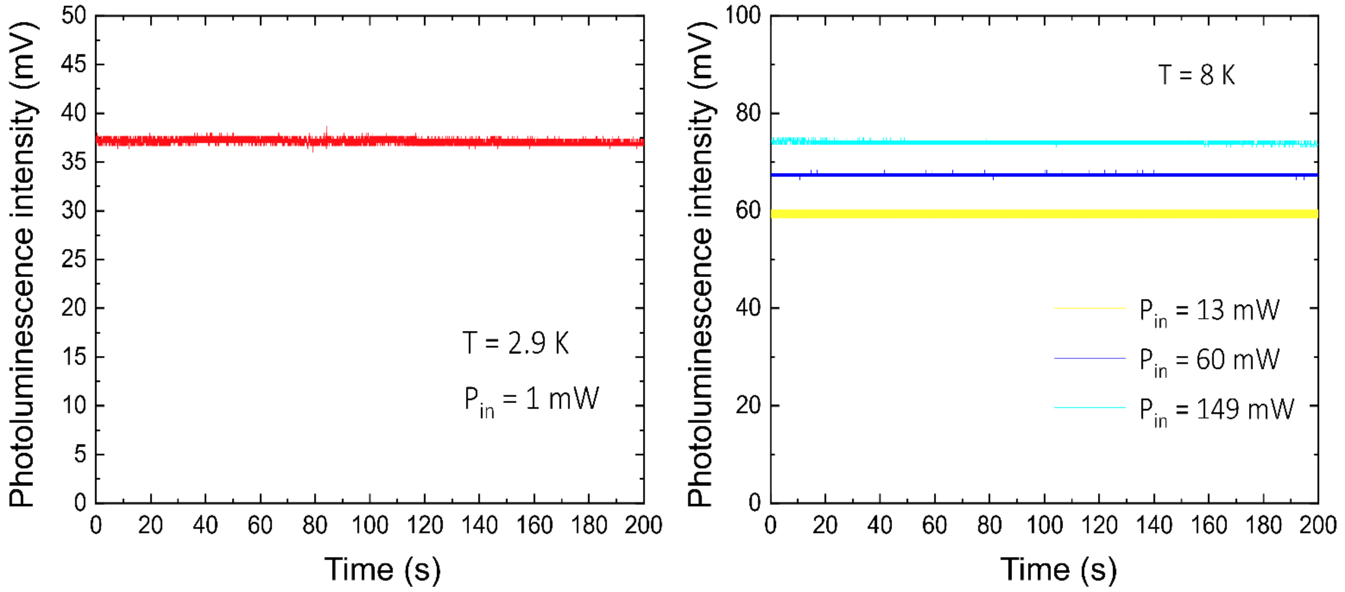

Supplementary Figure 14 | Photoluminescence intensity signal recorded over 200 s at different excitation powers and sample temperatures, showing the absence of photobleaching in [Eu_2Cl_6(4-picNO)$_4$(μ_2-4-picNO)$_2$]·$2H_2O$.

3.3.5. Homogeneous line broadening in [Eu_2]

Processes that cause broadening of Eu(III) in [Eu_2] are mainly attributed to low-energy molecular vibrations or rotations through 2-phonon processes[5,6]. In addition, nuclear spins carried by H or Cl elements could also induce broadening, although the linewidths observed in $EuCl_3$·6 H_2O are on the order of a few kHz. Spectral diffusion related to residual disorder caused by defects in the crystalline lattice (the so-called two-level systems, TLS) has also been observed in Eu(III) doped inorganic materials[5] and studied in detail in other systems such as single molecules embedded in organic crystals[7]. However, it should be noted that the narrow inhomogeneous linewidth observed in [Eu_2] does not point towards large disorder, but rather to a well crystallized system. In materials showing a comparable inhomogeneous linewidth, such as 60 nm Eu^{3+}:Y_2O_3 nanocrystals[8] or crystals with a high level of defects[9], the maximal broadening

due to disorder is about 50 kHz/K and thus significantly lower than what we observe. Only in amorphous materials, such as glasses, can Eu(III) show broadenings of 10s of MHz/K[10]. Here, the inhomogeneous linewidth is however 3000 GHz, much larger than what we measure in [Eu_2]. We also did not observe hole broadening as a function of the delay between burning and probing on a time scale up to 10 s, which suggests that slow spectral diffusion processes do not dominate the homogeneous linewidth. These comparisons should however be taken with caution as the nature of defects, their dynamics and their effects on Eu(III) in a crystal lattice composed of molecules such as [Eu_2] can be different than the ones encountered in inorganic systems. Planar defects—such as grain boundaries, stacking faults, and step edges—cause defects in molecular lattices. For example, a layered arrangement of molecules in the b×c plane was observed in the crystal lattice of [Eu_2] (Supplementary Figure S6). A possible occurrence of step edges between atomically flat layers could cause planar defects in the crystal lattice of [Eu_2] and occurrence of disorder related spectral diffusion. Further studies such as measuring dependence of homogeneous linewidth on temperature would be needed to clarify the different contributions. Studies as a function of applied magnetic fields could also identify possible contributions from impurities or defects carrying electron spins, while Eu-Eu interactions could be probed by using mixed Y-Eu samples[11]. Both lower temperature and high magnetic field could reduce Eu(III) homogeneous broadening and hence improve the quantum properties of [Eu_2] for applications in quantum technologies. However, current values do not prevent the coherent optical manipulation of spins using two-color pulses[12]. However, the fidelity with which spins can be controlled depends on the optical T_2. As a figure of merit, one can compare it to the shortest optical pulse that can be applied to Eu(III) and which is limited by the hyperfine splittings. In ^{153}Eu, these are typically in the order of 80 MHz, giving a minimal pulse duration *t* of about 12.5 ns and a maximal fidelity $\exp(-t/T_2) = 42\%$. This is lower than what has been achieved in inorganic crystals (96%)[13]. An improved molecular design and more favourable experimental conditions (lower temperature, high magnetic field) could increase T_2 and therefore achievable fidelities.

4. Supplementary references

1. Sheldrick, G. M. *SHELXT* – Integrated space-group and crystal-structure determination. *Acta Crystallogr. Sect. Found. Adv.* **71**, 3–8 (2015).
2. Sheldrick, G. M. Crystal structure refinement with *SHELXL*. *Acta Crystallogr. Sect. C Struct. Chem.* **71**, 3–8 (2015).
3. Thompson, L. C. & Kuo, S. C. Structure and absorption and fluorescence spectra of $Eu(PyNO)_8(C1O_4)_3$. *J. Common Met.* **148**, 173–179 (1989).

4. Binnermans, K., Lanthanide-Based Luminescent Hybrid Materials, Chem. Rev. **109**, 9, 4283–4374 (2009).
5. Goldner, P., Ferrier, A. & Guillot-Noël, O. Rare Earth-Doped Crystals for Quantum Information Processing. in *Handbook on the Physics and Chemistry of Rare Earths* vol. 46 1–78 (Elsevier, 2015).
6. Riesen, H. & Krausz, E. Persistent spectral hole-burning, luminescence line narrowing and selective excitation spectroscopy of the *R* lines of Cr(III) tris(2,2'-bipyridine) in amorphous hosts. *J. Chem. Phys.* **97**, 7902–7910 (1992).
7. Naumov, A. V. Low-temperature spectroscopy of organic molecules in solid matrices: from the Shpol'skii effect to laser luminescent spectromicroscopy for all effectively emitting single molecules, *Phys.-Uspekhi* **56**, 605–622 (2013).
8. Perrot, A. *et al.* Narrow Optical Homogeneous Linewidths in Rare Earth Doped Nanocrystals. *Phys. Rev. Lett.* **111**, (2013).
9. Flinn, G. P. et al. Anomalous optical dephasing in crystalline Y2O3: Eu3+. J. Lumin. **58**, 374–379 (1994).
10. Macfarlane, R. M. & Shelby, R. M. Homogeneous line broadening of optical transitions of ions and molecules in glasses. J. Lumin. **36**, 179–207 (1987).
11. Kunkel, N. et al. Dephasing mechanisms of optical transitions in rare-earth-doped transparent ceramics. Phys. Rev. B **94**, 184301 (2016).
12. Serrano, D., Karlsson, J., Fossati, A., Ferrier, A. & Goldner, P. All-optical control of long-lived nuclear spins in rare-earth doped nanoparticles. Nat. Commun. 9, 2127 (2018).
13. Rippe, L., Julsgaard, B., Walther, A., Ying, Y. & Kröll, S. Experimental quantum-state tomography of a solid-state qubit. Phys. Rev. A 77, 022307 (2008).
